# Records of fleas (Siphonaptera) from Delaware

**DOI:** 10.1093/jme/tjae069

**Published:** 2024-05-17

**Authors:** Ashley C Kennedy, Wil S Winter, Alfred L Gardner, Neal Woodman, Scarlet A Shifflett, Sierra Redus, Jeffrey R Newcomer, Ralph P Eckerlin

**Affiliations:** Mosquito Control Section, Division of Fish and Wildlife, Delaware Department of Natural Resources and Environmental Control, Newark, DE 19702, USA; Mosquito Control Section, Division of Fish and Wildlife, Delaware Department of Natural Resources and Environmental Control, Newark, DE 19702, USA; Department of Entomology and Wildlife Ecology, University of Delaware, Newark, DE 19716, USA; Division of Mammals, MRC 108, National Museum of Natural History, Smithsonian Institution, Washington, DC 20013, USA; Division of Mammals, MRC 108, National Museum of Natural History, Smithsonian Institution, Washington, DC 20013, USA; Population Conservation Genetics Group, USGS Eastern Ecological Science Center at the Patuxent Research Reserve, Laurel, MD 20708, USA; Department of Entomology and Wildlife Ecology, University of Delaware, Newark, DE 19716, USA; Mosquito Control Section, Division of Fish and Wildlife, Delaware Department of Natural Resources and Environmental Control, Newark, DE 19702, USA; Wildlife Services, Animal and Plant Health Inspection Service, United States Department of Agriculture, Annapolis, MD 21409, USA; Natural Sciences Division, Northern Virginia Community College, Annandale, VA 22003, USA; Department of Vertebrate Zoology, National Museum of Natural History, Smithsonian Institution, Washington, DC 20013, USA

**Keywords:** ectoparasite, Ceratophyllidae, Ctenophthalmidae, Pulicidae, vector surveillance

## Abstract

We present an annotated checklist of fleas (Siphonaptera) known to occur in the state of Delaware based on an examination of Siphonaptera collections at the University of Delaware and the Carnegie Museum of Natural History, as well as new specimens of fleas we collected from wildlife, other hosts, and tick flags. We review published records and compile them herein with our new records, which include 3 species previously unreported from Delaware. With these additions, there are now 18 flea species from 19 avian and mammalian hosts documented from Delaware.

## Introduction

Delaware is a small state in the mid-Atlantic region of the eastern United States, comprising 3 counties (New Castle, Kent, and Sussex, from north to south). Research on fleas (Siphonaptera) in Delaware has been at a standstill for several decades. The most recent publication pertaining to fleas in Delaware was that of Tindall and Darsie (1961), and only 15 species had been reported from Delaware as of 2023. This low number of species likely reflects limited sampling effort rather than an actual lack of flea diversity in Delaware, which is home to many suitable wildlife hosts and habitats for fleas.

To obtain a better understanding of the siphonapteran taxa in Delaware and their potential to impact human health, we collected fleas while sampling for ticks (Arachnida: Ixodida) from 2020 to 2023 as part of an ongoing statewide tick surveillance program. Surveillance activities included dragging vegetation along trails, trapping small and meso-mammals, and examining bird nests and road-killed mammals. In addition, we conducted a comprehensive search of the literature, and we examined preserved flea specimens in 2 major insect collections and corresponded with curators in an additional major insect collection.

## Field Sites

We conducted focused small mammal trapping for ectoparasites at 3 sites representing each of the 3 counties in Delaware:

Newark, New Castle Co. (ca. 39°35ʹ22″ N, 75°44ʹ41″ W) 2–3 November 2022; 29–30 June 2023; 6–7 July 2023; 17–19 July 2023; and 26–27 July 2023. Traps were deployed in line transects in forest edge and grassland edge habitats.

Killens Pond State Park, Felton, Kent Co. (ca. 38°59ʹ04″ N, 75°32ʹ07″ W) 16–19 May 2023. Survey transects for small mammals were obtained from several sites along the southern, western, and northwestern sides of Killens Pond. Habitats included stream edge, mature secondary forest, grassland, and pond-edge swamp forest.

Cape Henlopen State Park, Lewes, Sussex Co. (ca. 38°46ʹ15″ N, 75°05ʹ38″ W) 29 October–1 November 2023. Traps were deployed in traditional line transects in habitats that included secondary forest, forest edge, and marsh edge.

## Materials and Methods

We collected fleas directly from tick flags with forceps whenever they were observed while dragging vegetation during tick surveillance efforts. We also collected fleas directly from an abandoned Barn Swallow (*Hirundo rustica*) nest in Milford, Kent Co., after the breeding season had ended.

We captured small mammals with Sherman live traps (H.B. Sherman Traps, Tallahassee, FL), Museum Special snap traps (Woodstream Corporation Inc., Lancaster, PA, USA; 14 cm × 7 cm/69 mm × 141 mm), Victor Mouse traps (Woodstream Corporation Inc., Lancaster, PA, USA; 8.6 cm × 17.7 cm/49 mm × 99 mm), and pitfall traps (18 cm × 10 cm-diameter plastic containers) in accordance with Delaware Division of Fish and Wildlife permit no. 2023-WSC-032. We used a combination of oats, sunflower seeds, and peanut butter as bait and provided cotton balls in the Sherman traps as nesting material. We used Tomahawk traps (Tomahawk Live Traps, Hazelhurst, WI, USA) to catch squirrels. Protocols for collecting and processing mammals were approved by the Animal Care and Use Committee of the US National Museum of Natural History, Washington, and followed guidelines established by the American Society of Mammalogists for the use of wild mammals in research and education (Sikes et al. 2016). Small mammal host vouchers are deposited in the collection of the Division of Mammals, National Museum of Natural History, Smithsonian Institution, Washington, DC, USA, and Yale Peabody Museum, New Haven, CT, USA.

We obtained road-killed mammals when they could be accessed safely, and we collected ectoparasites from mesocarnivores (i.e., raccoons, *Procyon lotor*; red foxes, *Vulpes vulpes fulva*; and gray foxes, *Urocyon cinereoargenteus*) that were trapped and removed from public lands to protect nesting areas of threatened and endangered shorebirds during the shorebird breeding season under Delaware Department of Natural Resources and Environmental Control Nuisance Wildlife Control Operator Permit #095. We combed mesocarnivores with a flea comb over a white sheet and brushed all small mammals with a toothbrush over a white enamel pan.

We used forceps to handle fleas, which we preserved in vials of 95% ethanol. Fleas were processed for identification by partial clearing in a 10% KOH solution overnight, washed, dehydrated in an ethanol series, further cleared in xylene, and then mounted on microscope slides in Canada balsam. We identified them under a compound microscope using Benton’s (1983) taxonomic key.

To provide a comprehensive list of species, we compiled previous records from the scientific literature using a combination of print and digital resources such as JSTOR (Ithaka Harbors, Inc., New York) and Google Scholar (Google, Inc., Mountain View, CA, USA). We also examined the Siphonaptera in the Entomology Collection at the University of Delaware, Newark (UDCC), and the Robert Traub Flea Collection, Carnegie Museum of Natural History, Pittsburgh, for unreported specimens collected in Delaware. We corresponded by email with curators of the Florida State Collection of Arthropods, Gainesville (FSCA), the repository of the renowned Allen Benton Flea Collection, regarding specimens collected in Delaware.

We use the ESA-approved common names for fleas where available. For those species lacking approved names, the common names we use are presented in quotation marks.

## Results

### Family Pulicidae

#### *Pulex irritans* Linnaeus

*Old records.* Tindall and Darsie (1961) were the first to report the human flea from Delaware, noting that 3 ♂ and 5 ♀ were collected by D. MacCreary in an abandoned poultry house in Kent Co. in 1960. Benton (1980) listed *Pulex* sp. from Kent Co., presumably referring to the same collection event.

#### *Ctenocephalides canis* (Curtis)

*Old records.* The report of Trembley and Bishopp (1940) was the first record of the dog flea from Delaware but did not provide specific locality information. Likewise, Milliron (1958:42) did not give locality details while reporting that *C. canis* occurs statewide and was “annoying to man and other animals.” Tindall and Darsie (1961) reported this species from domestic or feral dogs (*Canis familiaris*) but did not provide collection details.

#### *Ctenocephalides felis* (Bouché)

*Old records.* Milliron (1958:42) was the first to report the cat flea from Delaware, although he did not give specific locality information, simply writing that, along with the dog flea, it occurred “statewide” and was “annoying to man and other animals.” Tindall and Darsie (1961) reported the cat flea from *Homo sapiens* but did not provide collection details. We examined slide-mounted specimens from the UDCC that had been collected on 30 November 1950 from a human in New Castle Co. that could be the source for the Tindall and Darsie (1961) record.

*New records.* We collected 1 ♀ *C. felis* from an eastern gray squirrel (*Sciurus carolinensis*) in Kent Co. on 23 October 2021. We also collected *C. felis* from *V. vulpes fulva* in Sussex Co. (30 March 2022, 1 ♀; 6 April 2022, 2 ♀; 14 June 2022, 2 ♀; 24 February 2023, 1 ♀). We examined slide-mounted specimens in the UDCC that we believe were previously unreported. These include 1 **♂** and 2 ♀ from *C. familiaris* in New Castle Co. collected on 10 October 2006 (coll. K. Scogen), 1 ♀ from an unknown host in Sussex Co. collected on 30 October 2014 (coll. R. Moore), and 1 ♀ from a house cat (*Felis catus*) in Kent Co. collected on 3 November 2014 (coll. C. Stubbs). The collections from *V. vulpes fulva*, *S. carolinensis*, and *F. catus* apparently are new host records for Delaware.

#### *Cediopsylla simplex* (Baker)

*Old records.* MacCreary (1945) was the first to report this species, sometimes called the “rabbit flea,” from Delaware. The host was a woodchuck (*Marmota monax*), but MacCreary did not provide additional collection details. Florschutz and Darsie (1960) reported 1 ♀ collected in 1939 by MacCreary from an unidentified species of rabbit in Kent Co. (not reported in MacCreary’s 1945 publication), and 46 ♂ and 63 ♀ from eastern cottontails (*Sylvilagus floridanus*) they collected in New Castle Co. in 1958.

*New records.* We collected 1 ♀ from *V. vulpes fulva* in Sussex Co. on 24 February 2023. This constitutes both a new county record and a new host record for *C. simplex* in Delaware.

#### *Echidnophaga gallinacea* (Westwood)

*New records.* We examined 3 slide-mounted sticktight fleas in the UDCC that we believe were previously unreported. They were collected from *C. familiaris* in Sussex Co. on 12 November 1962 by Burbutis and Bray. The occurrence of *E. gallinacea* in Delaware was not unexpected, given that the flea has been reported from Pennsylvania (Holland and Benton 1968).

### Family Vermipsyllidae

#### *Chaetopsylla lotoris* (Stewart)

*New records.* We collected *C. lotoris* from *V. vulpes fulva* in Sussex Co. (17 March 2022, 1 **♂**; 6 April 2022, 1 ♂, 1 ♀; 24 February 2023, 1 ♂, 3 ♀). We also collected 1 ♀ from *U. cinereoargenteus* and 8 ♀ from *P. lotor*, both carnivores collected on 24 February 2023 in Sussex Co. The occurrence of *C. lotoris* in Delaware is unsurprising given that it is known from Maryland (Benton 1980) and Pennsylvania (Holland and Benton 1968). *P. lotor* is the primary host for this carnivore flea, and there are numerous reports of it infesting *U. cinereoargenteus*; however, *V. vulpes fulva* is an unusual host for *C. lotoris*.

### Family Ctenophthalmidae

#### *Ctenophthalmus pseudagyrtes* Baker

*Old records.* MacCreary (1945) was the first to report *C. pseudagyrtes* from Delaware, where he collected it from a variety of hosts: northern short-tailed shrew (*Blarina brevicauda*), eastern meadow vole (*Microtus pennsylvanicus*), woodland vole (*Microtus pinetorum*), white-footed mouse (*Peromyscus leucopus*), and *S. floridanus*. Although he did not provide locality data, we examined slide-mounted specimens in the UDCC that were collected in 1939 by MacCreary in Kent Co. that we believe are a partial basis for his records and those of Tindall and Darsie (1961).

*New records.* We collected *C. pseudagyrtes* from *B. brevicauda* in Kent Co. (17 May 2023, 1 ♂, 1 ♀; 18 May 2023, 2 ♂, 1 ♀; 19 May 2023, 1 ♀).

#### *Corrodopsylla curvata* (Rothschild)

*New records.* We collected 1 ♀ *C. curvata*, sometimes called the “shrew flea,” from a tick flag in New Castle Co. on 9 June 2022. Although we have no host records for Delaware, this species is predominantly associated with shrews of the genera *Blarina* and *Sorex*. The occurrence of this species in Delaware is not unexpected, given previous reports from New Jersey (Burbutis 1956) and Pennsylvania (Holland and Benton 1968).

#### *Doratopsylla blarinae* Fox

*Old records.* MacCreary (1945) was the first to report *D. blarinae* from Delaware, where he found it on *B. brevicauda*. Although he did not report the locality, Tindall and Darsie (1961) reported 1 ♂ collected in 1939 by MacCreary from a “shrew” in Kent Co. that we believe is the basis for his record. Tindall and Darsie (1961) also report 19 ♂ and 14 ♀ collected in 1959 from *B. brevicauda* and 1 ♂ and 1 ♀ from *P. leucopus* in New Castle Co.

*New records.* We collected 17 ♂ and 9 ♀ from *B. brevicauda* in Kent Co. between 17 and 19 May 2023.

#### *Epitedia wenmanni* Rothschild

*Old records.* MacCreary (1945) was the first to report *E. wenmanni* from Delaware, where he collected it on *P. leucopus*. Tindall and Darsie (1961) reported 1 ♀ collected in 1939 by MacCreary in New Castle Co. that we believe is the basis of MacCreary’s record. Tindall and Darsie (1961) also report 1 ♂ collected in 1940 by MacCreary from *M. pennsylvanicus*, and 2 ♂ and 8 ♀, collected on *P. leucopus* in 1959 by Tindall, also in New Castle Co.

#### *Stenoponia americana* (Baker)

*Old records.* MacCreary (1945) was the first to report *S. americana* from Delaware, where he found it on 2 host species, *M. pennsylvanicus* and *M. pinetorum*. Tindall and Darsie (1961) reported 1 ♀ from *B. brevicauda* and 1 ♂ from *Peromyscus*, collected by MacCreary in New Castle Co. in 1939. Oddly, MacCreary’s publication does not include these host records. Tindall and Darsie (1961) also reported 2 ♂ and 5 ♀ collected in 1959 from *P. leucopus* in New Castle Co.

### Family Ceratophyllidae

#### *Ceratophyllus gallinae* (Schrank)

*Old records.* MacCreary and Catts (1954) were the first to document the European chicken flea, *C. gallinae*, from Delaware when they reported a large infestation on chickens (*Gallus gallus*) at a poultry farm in New Castle Co. This record was repeated by Tindall and Darsie (1961). At that time, Delaware constituted the southernmost extent of this species’ range in the United States. We found 15 slide-mounted *C. gallinae* in the UDCC, collected in August–September 1950 by MacCreary. This species was the first flea reported from an avian host in Delaware; all previous reports were based on fleas from mammalian hosts.

*New records.* We collected more than 100 *C. gallinae* from a *H. rustica* nest in Milford, Kent Co., on 13 October 2021. We slide-mounted 9 ♀ and 7 ♂ to retain as voucher specimens. They represent a new county record and a new host record for this species in Delaware.

#### *Ceratophyllus idius* Jordan and Rothschild

*Old records.* This species, sometimes called the “Purple Martin flea,” was reported from New Castle Co. by Benton (1980). We lack definitive information on the source of this record; however, we believe it is related to a call by Benton and Shatrau (1965:77) to “people interested in birds” to send them fleas collected from birdhouses. Allen Benton had previously placed a note in *Purple Martin News* asking for nests to be sent to him after birds had finished breeding in them. He received hundreds of nests from across the United States in the mail and identified and recorded many bird fleas from them (L.A. Durden, personal communication).

#### *Megabothris asio asio* (Baker)

*Old records.* MacCreary (1945) reported this species from *M. pennsylvanicus*, but did not specify where the host was collected. Florschutz and Darsie (1960) reported 1 ♂ collected by MacCreary in Delaware City, New Castle Co., and another 1 ♂ in New Castle, New Castle Co., both in 1939, and we believe these are the bases for MacCreary’s record. An unnumbered slide of *M. asio asio* from Delaware was located in the CMNH collection. The 2 fleas were 1 ♂ and 1 ♀, labeled, “ex meadow mouse, Delaware, New Castle County, Port Penn, 3 May 1939, D. MacCreary.” Presumably, the “meadow mouse” was *M. pennsylvanicus*. Holland (1950) reported *M. asio asio* from Kent Co. from the same host species, repeated MacCreary’s Delaware City record, and noted that Delaware constituted the southernmost record for the eastern subspecies in the United States.

#### *Orchopeas howardi* (Baker)

*Old records.* MacCreary (1945) reported this species under its junior synonym, *O. wickhami*, from the American red squirrel (*Tamiasciurus hudsonicus*), but did not report where it was collected. The CMNH has a slide (CMNH679320) with 1 ♂ and 1 ♀ *O. howardi* (ex. *T. hudsonicus*, DE, Newark, D. MacCreary, 1941); we believe this to be the basis of MacCreary’s record. Subsequent publications (Tindall and Darsie 1961, Benton 1980) report *O. howardi* from both New Castle and Kent counties. Tindall and Darsie (1961) reported 2 ♂ from *T. hudsonicus* in New Castle Co., and 9 ♀ and 5 ♂ from *S. carolinensis* in Kent Co.

*New records:* We collected this species from tick flags in all 3 counties: New Castle (13 June 2022, 1 ♀), Kent (1 November 2021, 1 ♀), and Sussex (11 May 2022, 1 ♀; 25 June 2022, 1 ♀). We also obtained it from several mammalian hosts. These included *H. sapiens* in New Castle Co. (30 May 2022, 1 ♀), which represents a new host record for this species in Delaware; *S. carolinensis* in Sussex Co. (10 September 2021, 1 ♀; 1 April 2022, 1 ♂, 2 ♀; 6 April 2022, 2 ♂, 2 ♀) and in New Castle Co. (22 October 2022, 1 ♀; 5 November 2022, 1 ♂); and Delmarva fox squirrel (*Sciurus niger cinereus*) in Sussex Co. (22 March 2022, 1 ♂, 2 ♀; 4 April 2022, 1 ♂; 10 June 2022, 1 ♂, 1 ♀; 6 October 2022, 1 ♂; 23 March 2023, 1 ♀). These collections are the first records of this species in Sussex Co. We also found previously unreported specimens in the UDCC, consisting of 1 ♂ from an unspecified nest in New Castle Co. (7 January 1965) and 1 ♂ from an unidentified squirrel in New Castle Co. (15 October 1982).

#### *Orchopeas leucopus* (Baker)

*Old records.* MacCreary (1945) was the first to report *O. leucopus* from Delaware, where he found it on 2 hosts, *M. pinetorum* and *P. leucopus*. Tindall and Darsie (1961) reported 1 ♂ and 2 ♀ collected by MacCreary from *M. pinetorum* in Kent Co. in 1939 that we believe is the basis for MacCreary’s record for that species. Tindall and Darsie (1961) also reported numerous specimens (10 ♂, 26 ♀) from *P. leucopus* collected in 1959 and 1 ♀ from a Carolina wren (*Thryothorus ludovicianus*) nest in 1960. They did not provide locality data for these collections; however, we examined slide-mounted specimens collected in 1959 in New Castle Co. by Tindall that we believe is the basis for his mouse record.

*New records.* We collected 1 ♂ and 1 ♀ from *P. leucopus* in New Castle Co. (3 November 2022) and another 1 ♂ and 1 ♀ from *P. leucopus* in Kent Co. (18 May 2023).

#### *Oropsylla arctomys* (Baker)

*Old records.* MacCreary (1945) was the first to report this species from Delaware, from 2 hosts, *V. vulpes fulva* and *M. monax*. Although he did not provide locality data, Florschutz and Darsie (1960) reported 1 ♀ from *M. monax* collected by MacCreary in New Castle Co. in 1939 that we believe is the basis of his record. Florschutz and Darsie (1960) reported 1 ♀ from a Virginia opossum (*Didelphis virginiana*) collected by Florschutz in 1958 in New Castle Co., noting that this is an unusual host for this species.

*New records.* We examined 1 ♂ from UDCC collected by R. Vair from *M. monax* in New Castle Co. on 8 April 1965. We believe this specimen to be previously unreported.

### Family Leptopsyllidae

#### *Odontopsyllus multispinosus* (Baker)

*Old records.* Florschutz and Darsie (1960) were the first to report *O. multispinosus* from Delaware, where they found it in 1958 on *S. floridanus* in New Castle Co. Tindall and Darsie (1961) listed *D. virginiana* as a Delaware host for this species, but did not provide collection data.

## Discussion

With the addition of 3 species (*C. curvata*, *E. gallinacea*, and *C. lotoris*), the total number of flea species reported from Delaware is now 18 (Table 1). This flea diversity is low compared to neighboring states. There are 25 flea species known from New Jersey (Burbutis 1956, Harlan and Kramer 1979), 31 from Maryland (Eckerlin 2011), and 32 from Pennsylvania (Holland and Benton 1968). Although a small state, the low diversity in Delaware more likely reflects a lack of sampling. For example, siphonapterans are currently documented from only 13 of the 33 species of native mammals in the state and from an even lower proportion of native avians. We expect that, with more targeted sampling, as many as 21 additional species may be reported from Delaware in the future based on their known distribution in adjacent states and the presence of suitable host species (Table 2). Of note, one specimen of *Xenopsylla cheopsis* (Rothschild), collected by E.P. Catts in Newark, New Castle Co. in 1960, is present in the FSCA; however, it is labeled as a laboratory-reared specimen and thus does not constitute a state record.

**Table 1.** List of species of Siphonaptera known from Delaware, indicating their county distributions in the state and occurrence in surrounding states

Taxon	New Castle	Kent	Sussex	NJ	PA	MD
Family Pulicidae						
* Pulex irritans*		X		X		
* Ctenocephalides canis*	X	X	X	X	X	X
* Ctenocephalides felis*	X	X	X	X	X	X
* Cediopsylla simplex*	X	X	X	X	X	X
* Echidnophaga gallinacea*			X		X	
Family Vermipsyllidae						
* Chaetopsylla lotoris*			X		X	X
Family Ctenophthalmidae						
* Ctenophthalmus pseudagyrtes*		X		X	X	X
* Corrodopsylla curvata*	X			X	X	
* Doratopsylla blarinae*		X		X	X	X
* Epitedia wenmanni*	X			X	X	X
* Stenoponia americana*	X			X	X	X
Family Ceratophyllidae						
* Ceratophyllus gallinae*	X	X		X		
* Ceratophyllus idius*	X					X
* Megabothris asio asio*	X	X		X	X	
* Orchopeas howardi*	X	X	X	X	X	X
* Orchopeas leucopus*	X	X		X	X	X
* Oropsylla arctomys*	X			X	X	
Family Leptopsyllidae						
* Odontopsyllus multispinosus*	X			X	X	X

**Table 2.** List of species of Siphonaptera known from adjacent states and not yet reported from Delaware

Taxon	State(s)
Family Pulicidae	
* Pulex simulans* Baker	New Jersey (Harlan and Kramer 1979)
* Cediopsylla inaequalis* (Baker)	New Jersey (Burbutis 1956)
* Xenopsylla cheopsis* (Rothschild)	Maryland (Luttermoser 1936); New Jersey (Burbutis 1956); Pennsylvania (Benton 1980)
Family Ctenophthalmidae	
* Conorhinopsylla stanfordi* Stewart	Maryland (Eckerlin 2011); New Jersey (Burbutis 1956); Pennsylvania (Holland and Benton 1968)
* Epitedia faceta* (Rothschild)	Maryland (Benton 1980); Pennsylvania (Holland and Benton 1968)
* Rhadinopsylla orama* Smit	Maryland (Fox 1940); Pennsylvania (Holland and Benton 1968)
* Tamiophila grandis* (Rothschild)	Maryland (Eckerlin 2011); Pennsylvania (Holland and Benton 1968)
* Nearctopsylla genalis* (Baker)	Pennsylvania (Holland and Benton 1968)
Family Hystrichopsyllidae	
* Hystrichopsylla tahavuana* Jordan	Pennsylvania (Holland and Benton 1968)
* Atyphloceras bishopi* Jordan	Pennsylvania (Holland and Benton 1968); New Jersey (Burbutis 1956)
Family Ceratophyllidae	
* Ceratophyllus riparius* Jordan and Rothschild *Nosopsyllus fasciatus* (Bosc)	Maryland (Luttermoser 1936); New Jersey (Burbutis 1956); Pennsylvania (Holland and Benton 1968) Maryland (Benton 1980)
* Opisodasys pseudarctomys* (Baker)	Maryland (Benton 1980); Pennsylvania (Holland and Benton 1968)
* Megabothris acerbus* (Jordan)	Pennsylvania (Holland and Benton 1968)
Family Leptopsyllidae	
* Leptopsylla segnis* (Schonherr)	Maryland (Yeh and Davis 1950)
* Peromyscopsylla catatina* (Jordan)	Maryland (Fox 1940); Pennsylvania (Holland and Benton 1968)
* Peromyscopsylla hamifer* (Rothschild)	Maryland (Johnson and Traub 1954); Pennsylvania (Holland and Benton 1968)
* Peromyscopsylla hesperomys* (Baker)	Maryland (Benton 1980); Pennsylvania (Holland and Benton 1968)
* Peromyscopsylla scotti* Fox	Maryland (Johnson and Traub 1954); New Jersey (Burbutis 1956)
Family Ischnopsyllidae	
* Myodopsylla insignis* (Rothschild)	Maryland (Fox 1940); New Jersey (Burbutis 1956); Pennsylvania (Holland and Benton 1968)
* Nycteridopsylla chapini* Jordan	Maryland (Jordan 1928)

Species occurring in neighboring states but not expected in Delaware due to lack of suitable hosts or habitat include *Orchopeas pennsylvanicus* and *Epitedia cavernicola* (both ectoparasites of the Allegheny woodrat, *Neotoma magister*, which is not found in Delaware), *Polygenis gwyni* (an ectoparasite of the hispid cotton rat, *Sigmodon hispidus*, likewise not found in Delaware), and *Catallagia borealis* (associated with mammals in boreal habitats, also not present in Delaware) (Fox 1940, Holland and Benton 1968, Harlan and Kramer 1979, Eckerlin 2011). Burbutis and Mangold (1956) report *Euhoplopsyllus affinis* (Baker) from New Jersey but note that it was likely brought into the state by imported rabbits and was not known to be established; we have no reason to suspect it would occur in Delaware.

We observed that flea species which are considered host specialists may feed on alternative hosts, e.g., the raccoon specialist *C. lotoris* feeding on red foxes. This polyphagous behavior introduces the potential for pathogen spillover. Some pathogen associations are known for flea species reported in Delaware. *X. cheopsis* Rothschild is the primary vector of *Rickettsia typhi*, the agent of murine typhus (Dobler and Pfeffer 2011), but *C. felis*, *E. gallinacea*, and *P. irritans* also act as vectors, and *C. felis* is an increasingly important vector in the United States (Blanton 2021). *C. felis* is also the primary vector and reservoir of *Rickettsia felis*, another agent of rickettsiosis (Brown and Macaluso 2016), and a vector of *Bartonella henselae*, an agent of bartonellosis or cat scratch disease (Chomel et al. 1996). *C. canis*, *C. felis*, and *P. irritans* can serve as intermediate hosts of the dog tapeworm, *Dipylidium caninum*. Although infestations are uncommon in humans, humans can be accidental hosts for this cestode (Dobler and Pfeffer 2011).
